# Transcriptional Autoregulatory Loops Are Highly Conserved in Vertebrate Evolution

**DOI:** 10.1371/journal.pone.0003210

**Published:** 2008-09-15

**Authors:** Szymon M. Kiełbasa, Martin Vingron

**Affiliations:** Max Planck Institute for Molecular Genetics, Berlin, Germany; Center for Genomic Regulation, Spain

## Abstract

**Background:**

Feedback loops are the simplest building blocks of transcriptional regulatory networks and therefore their behavior in the course of evolution is of prime interest.

**Methodology:**

We address the question of enrichment of the number of autoregulatory feedback loops in higher organisms. First, based on predicted autoregulatory binding sites we count the number of autoregulatory loops. We compare it to estimates obtained either by assuming that each (conserved) gene has the same chance to be a target of a given factor or by assuming that each conserved sequence position has an equal chance to be a binding site of the factor.

**Conclusions:**

We demonstrate that the numbers of putative autoregulatory loops conserved between human and fugu, danio or chicken are significantly higher than expected. Moreover we show, that conserved autoregulatory binding sites cluster close to the factors' starts of transcription. We conclude, that transcriptional autoregulatory feedback loops constitute a core transcriptional network motif and their conservation has been maintained in higher vertebrate organism evolution.

## Introduction

Network motifs are small patterns of interactions from which transcription regulation networks are built [1]. They are believed to carry out specific information-processing functions, so prediction of these circuits is a key step towards understanding the properties of living systems. Feedback, where the output signal of a network element influences its input signal, is common in regulatory networks. A transcriptional autoregulatory feedback loop is the simplest network motif built out of a transcription factor regulating its own transcription. In general, such a negative or positive feedback leads to nonlinear dynamics [2].

In higher organisms many examples of autoregulation have been reported often in the context of key cellular processes like development or differentiation. For example, Hes1 negative autoregulation plays an important role in neuronal differentiation in early chicken embryos [3]. PAX4 expression in human is activated during pancreatic development and then switched off by a strong negative autoregulatory effect [4]. It has been demonstrated that autoregulatory binding of E2F1 may provide a switch regulating accumulation of E2F activity during human cell cycle transitions [5]. Similarly, a positive autoregulation was demonstrated for serum response factor (SRF) in muscle gene expression [6]. The Ets-1 proto-oncogene is positively autoregulated by its own product [7]. Autoregulation of the Hoxa4 mouse gene is essential for maintaining normal levels of its expression during development of the embryo [8]. A model of direct positive autoregulation of Pax6 in mouse lens development has also been proposed [9].

The function of autoregulatory loops has been intensively studied. A synthetic circuit has been constructed to demonstrate experimentally that negative autoregulation can speed up transcription responses – a negative autoregulatory circuit approaches its steady-state value much faster than the non-autoregulatory circuit [10]. Additionally, it has been shown that such negative autoregulation speeds up the rise-time, but does not generally affect the turn-off time [10]. Moreover, negative autoregulation has been demonstrated to produce a gain of stability compared to non-autoregulatory system [11]. Experimental analysis has shown that self-repression decreases noise compared to expression from a non-regulated promoter [12]. Positive feedback is a mechanism that has been utilized to convert a graded input into a binary response in a eukaryotic gene circuit [13]. Mathematical modelling has been used to conclude that multiple stable states can only arise when a positive feedback loop is involved [14]. Bistability allows cells to maintain either of two distinct gene expression states, providing a mechanism by which past environmental conditions or intercellular signals can be remembered – this way a mixture of responses has been achieved in a single population with genetically regulated ratios [14]. Moreover, since bistable systems are expected to display some degree of hysteresis, its role as a mechanism acting as a buffer preventing noise to cause accidental switching between the states has been discussed [15].

Much of the work on understanding regulatory networks has focused on *Escherichia coli* and the yeast *Saccharomyces cerevisiae* for which protein-protein or protein-DNA interaction data are most abundant. It has been shown [16] that although similar transcriptional genetic circuits are found abundantly they do not share common ancestors - the transcriptional network motifs rather are a product of convergent evolution than circuit duplication. A similar analysis [17] has shown that although genes and interactions between them evolve by duplication, the network motifs themselves are not direct products of duplication with inheritance. Moreover, by combining interaction data with protein sequences it has been observed that orthologous transcription factors and their target genes share the same regulation provided that the protein sequences of the regulators are sufficiently conserved [18]. Such conserved relationships, forming a “regulog”, may be used to map interactions between different species. Moreover, the transcription factors have been found less conserved than target genes, suggesting that genes evolve slower than their regulation. In one of the largest studies, a comprehensive yeast transcriptional regulatory network has been constructed and analyzed [19]. The authors studied 157 transcription factors known to interact with more than 4000 target genes as determined in high-throughput chip-on-chip yeast experiments. They conclude that a few transcription factors are global regulators of many modules, while most of the factors control only a few modules.

Here we address the question of the importance of autoregulatory feedback loops in the course of evolution of higher organisms. We study a transcriptional regulatory network formed from the human genes and their interactions, which are predicted based on presence of strong putative transcription factor binding sites in regulatory regions of the genes. We look at overall properties of a subset of predicted human binding sites that are conserved down to chicken, fugu or danio and we concentrate on conservation of the simplest network motif. Since autoregulatory loops realize important biological functions we suspect that their number should be higher than expected and they should be conserved in the course of evolution.

In order to estimate the expected number of loops we introduce two different models to calculate autoregulation probability of a transcription factor. In the first model we assume, that each target has an equal chance to be regulated by a given factor, so the regulation probability can be expressed as a fraction of genes that have (conserved) binding sites predicted in own regulatory regions. We show that in this model indeed the amount of autoregulation is significantly higher than expected, but only when the whole regulatory network is taken into account. We do not observe this enrichment, when a part of the regulatory network limited only to transcription factors nodes is analyzed. We suspect, that this observation might be a consequence of higher conservation of transcription factors' regulatory regions. Therefore, we introduce a second autoregulation probability model, which takes into account the amount of conserved positions in gene regulatory regions. In this model we assume, that genes which have their regulatory regions more conserved have proportionally higher chance to be predicted as targets of a given factor. We demonstrate, that with respect to this model the amount of predicted conserved autoregulatory feedback loops is significantly higher than expected when conservation between human and chicken, danio or fugu is studied. We exclude the possibility that this growth is a consequence of unspecific binding sites which could arise if the transcription factors preferred sites of similar GC-content as observed in their own regulatory regions. In another test we show that our predicted autoregulatory binding sites cluster surprisingly close to the transcription start sites as expected for functional binding sites but not for falsely predicted ones. This positional preference cannot be explained by positional variations of other properties like GC-content or total amount of putative binding sites.

## Materials and Methods

This study is based on the genomic sequences, gene annotations and gene ortholog predictions available in the EnsEMBL database [20] version 46. Transcription factors and the position specific score matrices modeling sequences recognized by them were obtained from the Transfac 10.4 database [21].

### Genes and transcription factors

By *G_A_* we denote the set of all human genes annotated in the core version of the EnsEMBL database. |*G*| denotes the number of genes in the set *G*. By *G_F_* we describe genes that have been identified as transcription factors and for which profiles of recognized binding sites are available. Identification of transcription factors we perform using the ‘factor’ table of the Transfac database. First we map Transfac factor names to EnsEMBL human genes. For the factor names for which this procedure fails the rat and mouse parts of EnsEMBL are searched and human orthologs for the resulting genes are selected but only if they are uniquely mapped. Next, for each transcription factor, corresponding position specific count matrices (PSCMs) are extracted based on the information provided by the ‘matrix’ part of Transfac. This may result in one transcription factor being assigned to several PSCMs. To avoid introducing a bias due to possible higher representation of a factor with many associated matrices, we disambiguate this assignment by keeping for each transcription factor only the matrix with the highest information content.

### Regulatory regions

Prediction of transcription factor binding sites and their conservation is performed in two steps: first we scan the human DNA sequence for binding sites and next we check whether the predicted sites belong to a sequence fragment conserved with respect to the DNA sequence of another organism.

For each gene *g*∈*G_A_* we study sequence fragments from 3000 nt upstream till 3000 nt downstream around the start of the gene (without masking repeat regions or coding sequences). Transcription factor binding sites are predicted using the method described by [22] and as the authors suggest we choose for each PSCM a score threshold which corresponds to finding with a probability 0.05 a single false positive match every 500 tested positions of DNA sequence. By *s_f,g_* we denote the number of binding sites of a transcription factor *f*∈*G_F_* predicted within the DNA sequence around a gene *g*∈*G_A_*. Moreover, by *n_f,g_* we understand the number of positions around the gene scanned for a PSCM match.

Next, the predicted human binding sites are checked whether they belong to a sequence fragment conserved in another species close to the orthologue of a studied gene. We study conservation to rat, mouse, chicken, danio and fugu organisms separately. From the EnsEMBL Compara database we obtain lists of genes that are orthologs to the human genes and we use the BlastZ [23] algorithm to align each human gene DNA sequence to the DNA sequence of each of its orthologs. Before aligning we extend the sequences to 5000 nt upstream and 5000 nt downstream with respect to their transcription start sites. This way we calculate regions in the neighborhood of each gene from *G_A_* which are conserved in at least one of its orthologues from the chosen organism. Here we extend the above notation and introduce subsets 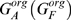 containing human genes (factors) which have an ortholog in organism *org*. For example, 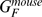 denotes the subset of human transcription factors which have at least one ortholog among mouse transcription factors. By 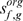 we describe the number of binding sites for factor *f* predicted in fragments of sequence around gene 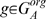. Similarly, 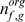 stands for the number of scanned positions of factor *f* binding within the conserved sequence part of gene *g*.

### Random models of autoregulation

The transcriptional regulatory interactions between transcription factors and their target genes can be presented as a directed graph. The nodes correspond to all genes *G_A_* and directed edges, always pointing from transcription factors *G_F_* to their target genes, are interpreted as “predicted to be regulated” relations. An edge *f*→*g* is added to the graph only when at least one binding site is predicted around the target gene, so when *s_f,g_*≥1.

An *autoregulatory feedback loop* is defined as at least one predicted binding site in a transcription factor's own regulatory region, or equivalently *s_f,f_*≥1 for *f*∈*G_F_*. An autoregulatory loop is represented by an edge in the graph pointing to the same transcription factor node from where it originates. Therefore, the number of autoregulatory loops obtained by prediction of binding sites can be written as:
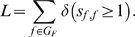



The hypothesis which we study is whether in the graph there are more predicted autoregulatory loops than expected. In order to estimate how probable it is to observe by chance *L* autoregulatory loops we first estimate probabilities for a transcription factor to be a target of itself. Then using these probabilities we simulate the total number of autoregulatory loops. We study separately the cases, when targets are defined as all genes *G_A_* or as transcription factors only *G_F_*. Moreover, the requirement that binding sites should be conserved causes some nodes or edges to disappear from the graph. Therefore, we study the above hypothesis for different conservation depths.

The simplest gene-oriented model to estimate the autoregulation probability *p_M_* (*f*; *G*) of a transcription factor *f*∈*G_F_* assumes that each gene (from *G*) has the same chance to be regulated. Then the probability would be estimated by a fraction of genes predicted to have a binding site of the factor *f*

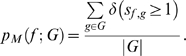



The second, site-oriented model of autoregulation takes into account the amount of conserved positions in gene regulatory regions. The site-oriented estimator of autoregulation probability *p_S_* (*f*; *G*) for each factor *f* takes into account the total number of binding sites in the genome 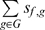 predicted over the total number of positions scanned for binding 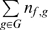. Using the hypergeometric distribution *p_H_* we calculate the probability of observing at least one predicted site among *n_f,f_* factor's positions in conserved sequence assuming that the predicted sites were randomly distributed over all genomic conserved positions
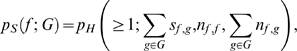
where *p_H_* (≥*c*; *a*,*b*,*N*) denotes the probability that there are at least *c* elements shared between two sets of sizes *a* and *b* whose elements were independently and randomly chosen from a set of *N* elements. Equivalently, the above formula can be written as:
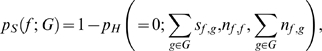
where *p_H_* ( = *c*; *a*,*b*,*N*) denotes the probability of exactly *c* elements to be shared between the sets of *a* and *b* elements chosen from a set of *N* elements.

Finally, once we have the probabilities of autoregulation for each transcription factor we may estimate the probability distribution of observing a given number of autoregulatory feedback loops. We perform 10^6^ simulations where we randomly assign an autoregulatory loop to a factor based on factor's individual autoregulation probability. This allows us to estimate p-values for observing a given number of autoregulatory loops, which we use to evaluate our hypothesis. The expected number of autoregulatory loops estimated from the set of genes *G* we denote by *L_M_* (*G*) or *L_S_* (*G*) depending which probability model is used. Moreover, 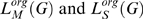 stand for expected averages calculated for genes and sequence fragments conserved between human and organism *org*.

## Results and Discussion

The choice of probability model is crucial in determining whether the number of autoregulatory loops in a predicted gene regulatory network is surprisingly high. Key function of such a probability model is to define the probability that a gene is regulated by a given transcription factor. We study a simple and a more sophisticated probability model.

In the simple model, when a transcription factor regulates a certain fraction of the genes, this number is also taken to be the probability that the factor regulates itself. Below it will be shown that this model suffers from a bias towards regulation of genes with a large part of their upstream sequence evolutionarily conserved, simply because it is more likely to predict a binding site within a longer sequence stretch than within a short one. Since, in particular, transcription factors tend to display high conservation of their upstream regions, this bias prevents us from recognizing particular topological features among transcription factors.

Due to the low specificity of binding site predictions, we cannot afford to drop the focus on conserved predicted binding sites. We thus put forward a more complex probability model that accounts for the amount of conserved upstream sequence. In this model, it is recognized that extensive upstream sequence conservation makes false positive predictions more likely and, consequently, the probability of true regulation needs to be downweighted. The probability for regulation can be computed as the probability of observing a non-zero overlap between all predicted binding sites and the ones upstream of the gene, always accounting for the amount of the conserved sequence. A hypergeometric distribution describes just this situation and the formulae are given in the Methods Section.

The key quantity needed to formalize the intuition of autoregulatory loops being frequent, is the number of autoregulatory loops in a network as a random variable. Each transcription factor contributes to this number with the probability that the factor, seen as a target gene, could be its own target, i.e. with its own probability to regulate a gene. Thus, one needs to add for all transcription factors these probabilities to obtain the expected number of autoregulatory loops. The Methods Section describes the simulations employed to determine the distributions for this quantity under the two probability models, respectively.

In total we extracted |*G_A_*| = 30793 human genes and corresponding regulatory sequences around their transcription start sites. Out of these genes |*G_F_*| = 292 have been identified as transcription factors and we could associate each one of them with at least one position specific count matrix (PSCM). The number of PSCMs annotated to a transcription factor varies as presented in the Table 1. For each transcription factor we choose the matrix of the highest information content, and afterwards, since several transcription factors are linked with the same PSCM the total number of different PSCMs is smaller than number of transcription factors and equals 217.

**Table 1:** Distribution of numbers of PSCMs associated with transcription factors.

Number of PSCMs	Count	Transcription factors
1	136	…
2	77	…
3	47	…
4	20	…
5	10	…
6	11	…
7	1	HNF4A
8	0	-
9	0	-
10	3	MEF2A, TCF3, CREB1
11	0	-
12	1	POU2F1
13	1	E2F1

In total 292 transcription factors, which could be mapped to human genes, are linked with 392 different PSCMs. In this study we choose for each transcription factor only the PSCM of the highest information content. This leads to 217 different PSCMs associated with the factors.

For all the genes we extracted corresponding orthologs in mouse, rat, chicken, fugu and danio genomes. Table 2 summarizes the numbers of human genes found to have at least one ortholog in a given species and the average lengths of fragments of regulatory regions which are conserved in the given species. Moreover, the numbers of transcription factors predicted to have an autoregulatory binding site are given. In general, for all analyzed species larger fractions of regulatory regions are conserved in the transcription factors *G_F_* than in all genes *G_A_*. As a consequence, the probabilities *p_M_* and *p_S_* differ depending on the set of genes used for their calculation.

**Table 2:** Numbers of conserved transcription factors and genes.

Organism *org*	Number of autoregulated factors (fraction [%]) *L^org^*	Number of factors 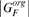	Sequence conservation around factors [%]	Number of genes 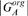	Sequence conservation around genes [%]
human	231 (100)	292	-	30793	-
human-mouse	156 (56.5)	276	65.2	18136	50.4
human-rat	135 (51.5)	262	56.5	16891	44.5
human-chicken	39 (24.2)	161	11.5	10658	8.0
human-fugu	34 (17.8)	191	8.3	10263	5.8
human-danio	31 (16.5)	188	7.6	10040	4.9

*_L_^org^* gives the numbers of autoregulatory loops observed by counting binding sites predicted in regulatory region fragments conserved between human and organism *org*. 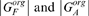 give numbers of human factors and genes which have orthologs in *org*. The remaining columns give an average fraction of gene regulatory regions that could be aligned with regulatory regions of the orthologs.

**Figure 1:**
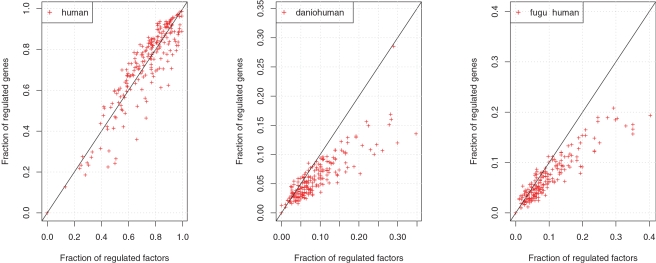
Comparison of fractions of genes and factors targeted by a transcription factor. Each point corresponds to a transcription factor *f*∈*G_F_*. Horizontal axes provide the fraction of factors 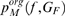 predicted to have a conserved binding site of *f*. Vertical axes give the fraction of regulated genes 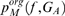. Three cases are shown: no conservation, conservation to danio and to fugu.

As shown in Figure 1 the majority of human genes are predicted to be regulated by each transcription factor even when strict thresholds for generation of a putative binding site are used. This is a well known problem related to low information content of PSCMs and long sequences studied, which leads to many false positive predictions.

Table 3 compares numbers of autoregulatory feedback loops obtained by counting site predictions *L^org^* for different levels of sequence conservation with expected numbers calculated based on the introduced probability models. Additionally, the probabilities of obtaining in the simulation at least as many autoregulatory loops as *L^org^* are given in parenthesis. We study these probabilities at the 0.05 significance threshold.

**Table 3:** Observed and expected numbers of autoregulatory loops.

*org*	*L^org^*	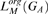	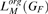	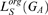	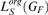
human	231	218.3 (0.034)	218.0 (0.034)	232.1 (0.61)	229.6 (0.45)
human-mouse	156	128.2 (0.0005)	149.0 (0.21)	153.4 (0.38)	148.5 (0.16)
human-rat	135	115.0 (0.0082)	131.4 (0.35)	129.1 (0.20)	125.5 (0.083)
human-chicken	39	27.7 (0.017)	39.6 (0.57)	23.4 (0.00006)	25.1 (0.00045)
human-fugu	34	20.1 (0.0019)	27.7 (0.12)	19.3 (0.00031)	20.8 (0.00062)
human-danio	31	17.6 (0.0014)	25.6 (0.15)	18.7 (0.0015)	17.6 (0.00031)

The *L^org^* column gives the number of conserved autoregulatory loops. Subsequent columns list expected numbers of conserved autoregulatory loops and p-values of the observed numbers for two different probability models *p_M_* and *p_S_* trained either only on the transcription factors *G_F_* or on all genes *G_A_*.

The counts *L^org^* are significantly higher than expected with respect to the gene-oriented probability model trained on all genes 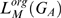. For all depths of conservation the model gives us strong evidence supporting our hypothesis. In particular, when conservation between human and the fishes is studied only about 55% of the autoregulatory loops could be explained by the model. But once the regulatory network is narrowed to the interactions predicted between transcription factors, the gene-oriented model 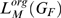 gives expectations no longer significantly different than the counts *L^org^*. This is a consequence of higher sequence conservation observed for the transcription factors than for average genes. Therefore, the hypothesis evaluated in the context of interactions exclusively between the transcription factors with respect to the gene-oriented model has no support.

In the site-based model the influence of varying sequence conservation is taken into account. For this model trained on all genes 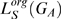 we observe that the counts are significantly higher than expectations for feedback loops conserved down to chicken, fugu and danio. On average, approx. 70% more autoregulatory loops are found than expected by the model. This provides strong evidence in favor for our hypothesis - among conserved regulatory regions transcription factors tend to have own binding sites more often than expected from the density of their sites in other targets. This observation is also valid, when the site-based model is trained only on the transcription factors. Here as well there appears to be significantly more feedback loops conserved down to chicken, fugu and danio compared to 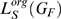.

**Figure 2:**
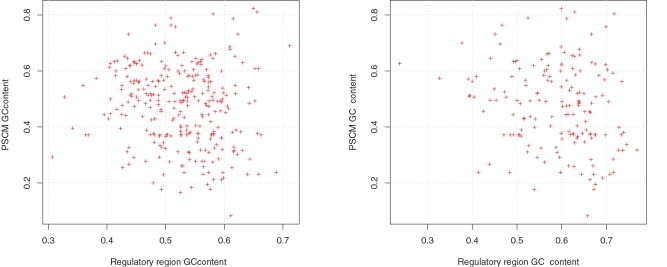
Relation between GC-contents of transcription factor's regulatory regions and corresponding PSCMs. Left: human sequences were used for regulatory region GC-content calculation. Right: fragments of human sequence conserved in fugu.

The procedure that we use for prediction of binding sites assumes the same background properties of all studied sequence positions. As a consequence, PSCMs with high GC-content would by chance occur more frequently in GC-rich regulatory regions. Therefore, we check whether GC-contents of transcription factors PSCMs and of corresponding regulatory regions correlate, which could explain predicted preferences of transcription factors to bind own regulatory regions. Figure 2 presents a scatter plots illustrating the dependence of both GC-contents when the complete regulatory regions are used or only their fragments conserved between human and fugu. We observe no correlation for any conservation depth. Therefore we conclude that unspecific GC-related preference of transcription factors does not explain overrepresentation of putative autoregulatory loops.

**Figure 3:**
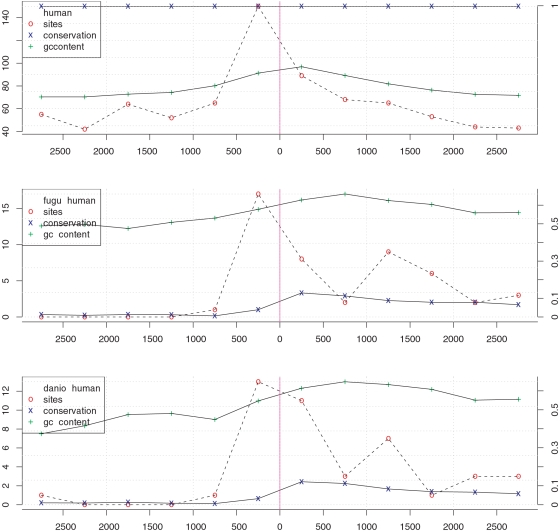
Positional distribution of autoregulatory binding sites. Amounts of conserved sequence and numbers of autoregulatory binding sites observed in bins located at different distances with respect to factors starts of transcription. Left axes present amount of contributing autoregulatory binding sites within a bin; right axes show fraction of conserved nucleotides in all factors within a bin. Top chart corresponds to the human sequence; below, for conserved sequence fragments to fugu and danio.

Figure 3 shows the positional behavior of conserved sequence fragments and predicted autoregulatory binding sites. We define bins of equal lengths located at different distances with respect to transcription start sites (TSSs) of the factors *G_F_*. Within each bin we count the amount of conserved nucleotides and average GC-content of the conserved sequence. Moreover, we calculate the number of autoregulatory binding sites predicted within a bin. For all shown depths of conservation a striking growth of the number of putative autoregulatory binding sites is observed in the bin located directly upstream of the TSSes. Moreover, autoregulatory binding sites are rather predicted downstream of TSSs than in the further upstream bins (excluding the first upstream bin). These distributions are significantly different from a uniform distribution that would be expected for random binding sites. A slight growth of amount of conserved sequence caused by higher conservation of exons shows a different shape and does not explain observed positional distribution of autoregulatory sites. Similarly, the average GC-content resembles the conserved sequence pattern and does not seem to influence autoregulatory sites distribution either.

In order to further test autoregulatory dependences between transcription factors and corresponding PSCMs we created a set of PSCMs with randomly shuffled positions. In general such a shuffling procedure should destroy a preference of a transcription factor to its own promoter. Indeed, as expected, the resulting number of predicted autoregulatory loops was no longer significantly different from the expected value.

Summarizing, we have shown that the number of autoregulatory feedback loops conserved between human and fugu, danio or chicken is significantly higher than expected in the site- and gene-oriented models. This significant overrepresentation we interpret as a consequence of biological importance of autoregulatory network motifs in regulation of processes maintained by cells. The predicted autoregulatory loops seem to constitute the core of conserved regulatory relationships across several distant species. The contributing sites have been shown to have positional preference towards annotated transcriptional start sites of the factors, which cannot be explained by a bias caused by GC content or non-uniform sequence conservation.
